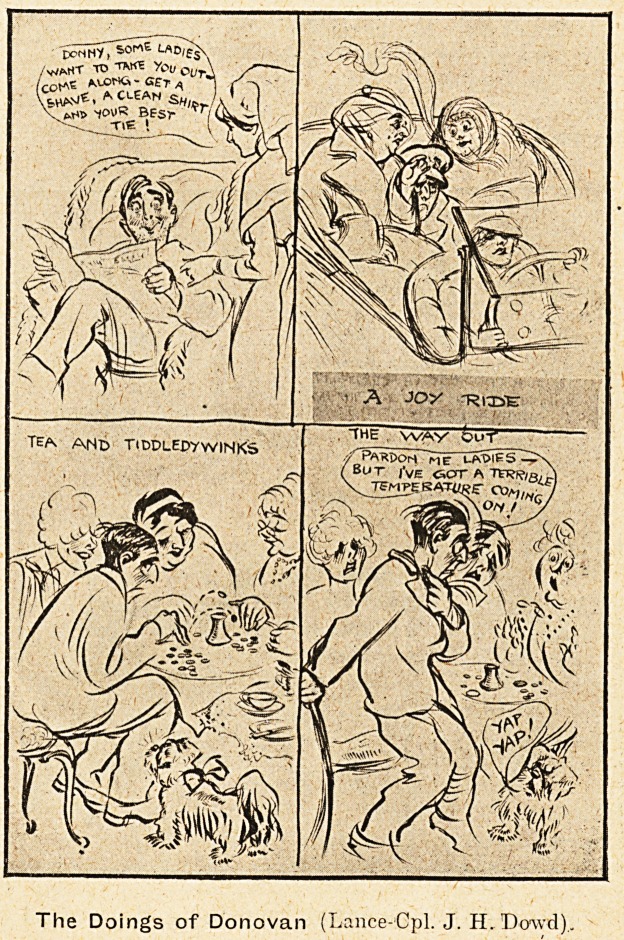# The Third London Artists and Their Work

**Published:** 1917-12-15

**Authors:** 


Waiting for Visitors (Cpl. Paul Kirk)

### TOMMY IN HOSPITAL.

### The Third London Artists and their Work.

It is not only the general public which, we hope, will take advantage of the exhibition of drawings now open at the Camera Club, 17 John Street, Adelphi. For though the public cannot but be interested in this amusing series by which the

Types of the British Army (Lance-Cpl. J. H. Dowd)

artists of the 3rd London General Hospital, Wandsworth, have made their hospital famous, hospital managers have much to learn from this exhibition. Many, if not all, of the black-and white drawings now on view have appeared in the 3rd London's *Gazette*. Therefore, our readers will have seen some of them, since the critical reviews which have appeared in The Hospital on the heels of each issue of the gazette will have failed of their purpose if they have not led our readers to turn to the gazette itself. It cannot be denied, however, that the drawings gain from being exhibited together. The spirit which one suggests is enforced by contiguity or contrast with another. The total effect resembles that of a massed brass band.

All, or nearly all, are humorous, and express the cheeriness of busy people, who would find the pressure of work and the restraint of institutional life unbearable if a saving sense of humour did not permeate their routine. It will be remembered that the staff of the 3rd London Hospital was recruited mainly from art students, and that the exuberance natural to such a company has infected the work of the place. In the drawings, therefore, we find, as we should expect, a certain recklessness and vigour which drive home, when the draughtsman

The Discovery (Lieut. J. A. Grant).

**TOMMY IN HOSPITAL—(*continued*).**

is skilful, the joke which forms the text of most of them.

To leave the Camera Club for the atmosphere of a dull London day is like awaking from a night-

Keep 'em Smilin' (Lance-Cpl. J. H. Dowd).

mare, one of those nightmares, not by any means disagreeable, but so called from the stress and unaccountability of the experience. Vigour is the prevailing note, and every drawing is an excitement. The lines sprawl and rush and concentrate, and the faces of the people are uncannily inhuman. It is the figure and the attitude which interest Lance-Corporal J. H. Dowd, the principal draughtsman. The faces are an exaggerated expression of pleasure, anger, excitement, or penitence. This is one proper method of caricature. And it is the chief characteristic of the work exhibited. The illustrations which we are privileged to reproduce give a too modest idea of the prevailing excitement. To go to the Camera Club is like a visit to "the movies," and to give this suggestion of kaleidoscopic motion is no small praise to the vigour of the work.

The few quiet drawings, like that of "Sleep," by Private E. R. Collings, and particularly that called "A Study," by Corporal T. Hobson Lobley, gain much by the contrast which they afford. So, in a sense, do the sentimental studies, like Sergeant Vernon Lorimer's "Memories," or Corporal Paul Kirk's "Waiting for Visitors," or Private Ogle's "How Bullecourt was Taken." Nor must the quiet humour of Lieutenant J. A. Grant's "A Pertinent Question" be missed. These show that the vigorous method is not the only one, even in caricature; and in caricature beauty, as a rule, is not aimed at. There can be no doubt, however, that Mr. C. R. W. Nevinson's drawings, which, in the style of the Cubists, represent a vigorous emphasis upon the planes in which light falls upon illuminated objects, are the best artistically in the collection. His work is now so well known that reproduction is unnecessary. His drawings achieve a beauty at which the others do not aim, and the Cubist method is unsurpassed in the vividness with which it endows even figures in repose. But this vigour is gained not by crude representation, but as a quality in the method itself.

It will be apparent from this brief notice that the lighter side of hospital life is vividly represented by this collection, and the only wonder is whether the exaggeration employed may not disguise the very fact which it was designed to emphasise—namely, the jolly spirit of good-fellowship which is the secret of the charm that hospital life brings to many. People who have spent their lives in hospital often say that they could not live in any other atmosphere, and this

The Doings of Donovan (Lance-Cpl. J. H. Dowd).

will be apparent to all who remember the attachment which school or university life has for all who have experienced it. The fun of it all is emphasised here.

But there is a practical lesson to be drawn from

**TOMMY IN HOSPITAL—** (*continued*).

this exhibition, which we hope that no hospital manager will miss. It is the power of the draughtsman as a maker of appeals. Why does not every hospital possess its own artist? Why have they been content to possess a publicity department, in which the draughtsman has no share? The faculty of drawing is not so rare as it is supposed to be. The truth is that that which is never looked for will never be found. An admirable example to all voluntary hospitals has been set by the Royal Hospital for Incurables at Putney Heath. Pictures and illustrations of a high order have long distinguished its appeals. Where the camera has led the way, the draughtsman should follow. We all know how letters of appeal are tucked away in the corners of the daily newspapers. We also know that these often make unnecessarily dull reading. Lord Knutsford, for instance, has done good work in an attempt to improve the standard. But this is not enough. A good drawing would arrest attention often better. In days when the drawing of advertisements has become a profession, there can be no excuse for not adopting the hospital poster. But the ideal should be for each hospital to produce its own draughtsman. This exhibition at the Camera Club has shown what one hospital has been able to accomplish. Its special advantages should only encourage others to make the most of their own. For, after all, it was only from small beginnings that the modern technique of written appeals has grown.

## Figures and Tables

**Figure f1:**
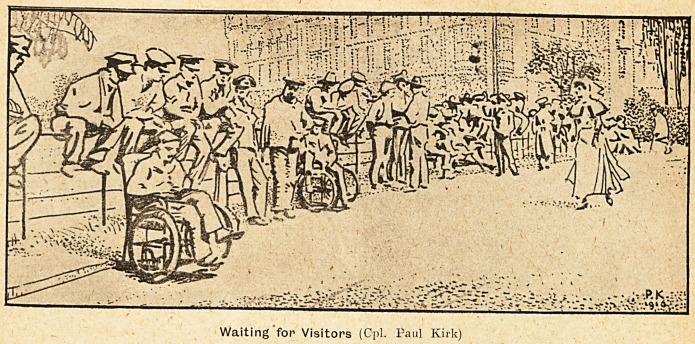


**Figure f2:**
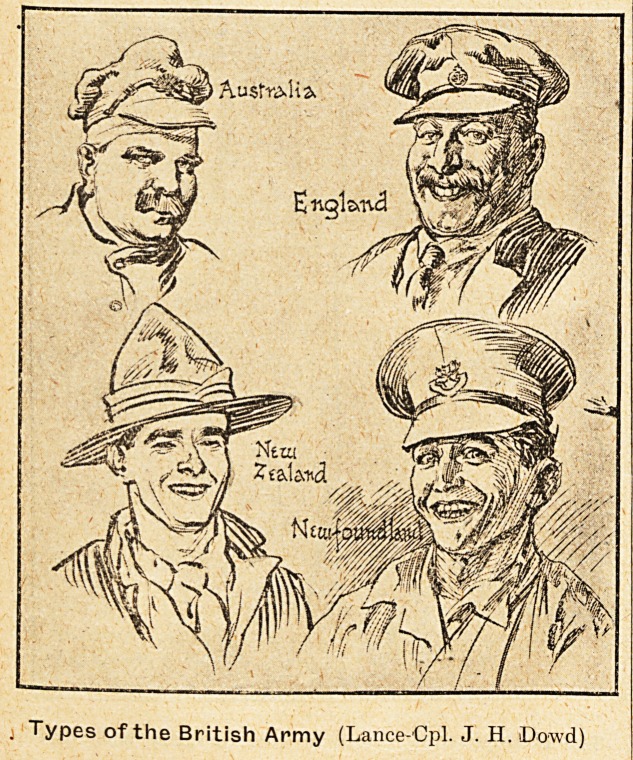


**Figure f3:**
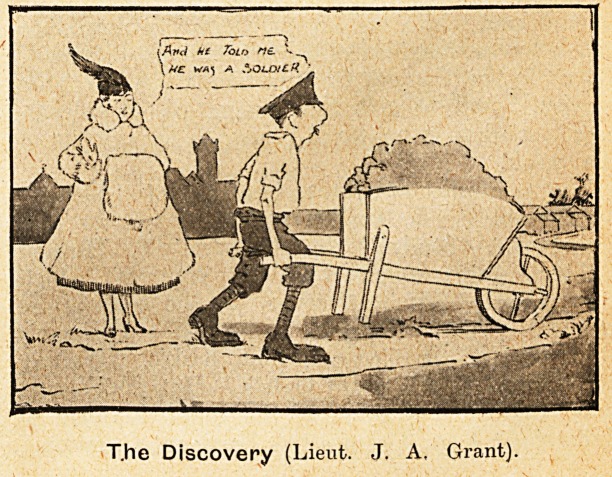


**Figure f4:**
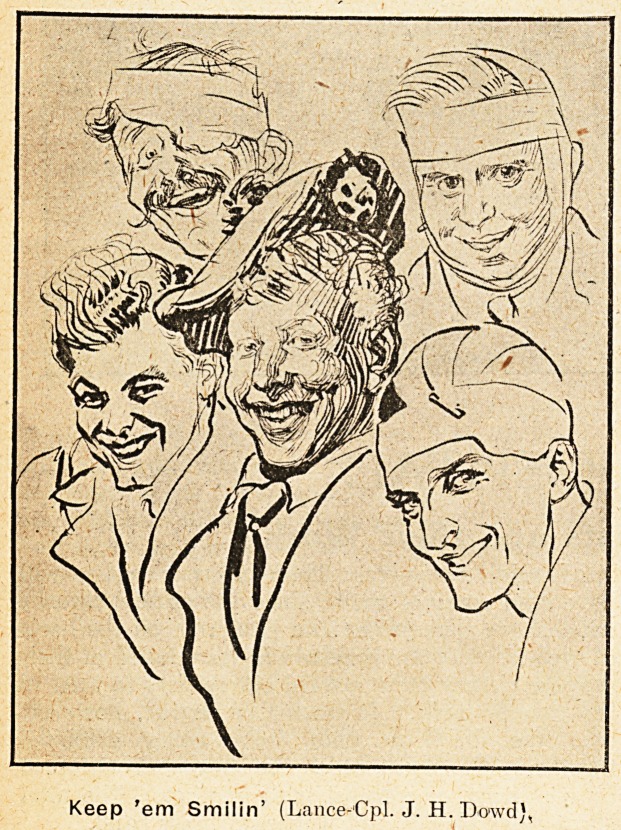


**Figure f5:**